# The Impact of Vaccination on COVID-19 Outcomes in Vietnam

**DOI:** 10.3390/diagnostics14242850

**Published:** 2024-12-18

**Authors:** Ngoc-Lan Thi Nguyen, Hien Thi Thu Nguyen, Vang Le-Quy, Thu-Ba To, Huy Thinh Tran, Tuan Duc Nguyen, Yen Hoang, Anh-Thu Nguyen, Lan Thi Phuong Dam, Nhat-Linh Nguyen, Anh Tuan Dinh-Xuan, Thanh-Van Ta

**Affiliations:** 1 Hanoi Medical University Hospital, Hanoi 100000, Vietnam; ngoclannguyen@hmu.edu.vn (N.-L.T.N.); tranhuythinh@hmu.edu.vn (H.T.T.); ductuan@hmu.edu.vn (T.D.N.); 2 Biochemistry Department, Hanoi Medical University, Hanoi 100000, Vietnam; nganhthu01@gmail.com (A.-T.N.); damphuonglan1988@gmail.com (L.T.P.D.); 3 Department of Molecular Diagnostics, Aalborg University Hospital, 9100 Aalborg, Denmark; hien@rn.dk; 4 AVSE Global Medical Translational Research Network, 75001 Paris, France; nguyenli@who.int; 5 Faculty of Information Technology, Duy Tan University, Danang 550000, Vietnam; vlequy@novodan.eu; 6 Novodan ApS, 9100 Aalborg, Denmark; 7 Insitut Galien Paris-Saclay, Université Paris-Saclay, 75001 Paris, France; thuba.to@avseglobal.org; 8 Department of Science and Technology Management, Hanoi Medical University, Hanoi 100000, Vietnam; yenhoang@hmu.edu.vn; 9 Department of Respiratory Medicine and Physiology, Hôpital Cochin, 75001 Paris, France

**Keywords:** COVID-19, prior vaccination, clinical outcome

## Abstract

**Objectives**: This study aimed to assess the effectiveness of the COVID-19 vaccine on the outcomes of patients in three hospitals in Vietnam. **Methods**: An observational study involved 3102 confirmed COVID-19 patients from Vietnam. Participants were classified into unvaccinated, partially vaccinated (one dose) (PV), fully vaccinated (two doses) (FV), and boosted (three doses) groups. We used a regression model to assess the relationship between vaccine status and disease outcome, including mortality, persistent symptoms after treatment, and hospital duration. **Results**: The proportions of unvaccinated, PV, FV, and boosted groups were 43.39%, 4.63%, 43.93%, and 8.05%, respectively, and 48% of the participants had at least one comorbidity. The proportion of severe clinical disease was significantly higher in the unvaccinated compared with the vaccinated. Biomarkers of cellular injury and organ failure, e.g., aspartate aminotransferase (AST), ferritin, troponin T, proBNP, D-dimer, and urea plasma concentration were significantly higher in unvaccinated and PV patients compared with FV and boosted patients. Age was the most crucial predictor of critical illness, followed by vaccine status, hypertension, diabetes, heart disease, and chronic kidney disease. The unvaccinated group had the highest proportion of deaths (5.2% vs. 1.4% and 0.3% in FV and boosted groups, respectively). **Conclusions**: Vaccination reduced mortality and both hospitalization length and disease severity in COVID-19 survivors, especially the older and patients with chronic comorbidities.

## 1. Introduction

At the end of 2019, the emergence of SARS-CoV-2, which causes acute respiratory distress syndrome, sparked a pandemic of a dangerous infectious disease globally [1,2,3]. This virus resulted in millions of cases and deaths, and health-care systems were under immense pressure to manage the pandemic [4,5]. Individuals infected with SARS-CoV-2 may exhibit various clinical symptoms, ranging from no symptoms to severe illness or even death. The severity of the illness is generally classified into five groups: (i) asymptomatic/presymptomatic infection, (ii) mild illness, (iii) moderate illness, (iv) severe illness, and (v) critical illness [6]. The worst-case scenario of COVID-19 can be acute respiratory distress syndrome (ARDS) and multiorgan failure.

One year after the outbreak, the availability of approved vaccines had alleviated the pandemic by successfully preventing COVID-19 propagation in society and effectively reducing the severity and mortality in hospitalized patients [7,8,9]. However, the emergence of new virus variants such as Delta and Omicron raised concerns about their transmissibility, waning vaccine-induced immunity, and variant immune evasion [10]. An additional vaccine (the third dose) was recommended to protect against the new variants. Various studies have shown that booster doses can maintain the immunity response at high levels, especially among vulnerable populations. For example, a retrospective cohort study in Italy reported that a second booster dose of mRNA vaccine administered 14–118 days after the initial administration is moderately effective in preventing SARS-CoV-2 infection among individuals aged ≥80 years, particularly during periods when Omicron variants are predominantly circulating [11]. Another study conducted on 222,772 emergency department and urgent care encounters and 87,904 hospitalizations among COVID-19-infected adults in the United States found that during the periods of Delta and Omicron predominance, the receipt of a third vaccine dose reduced the cases of COVID-19-associated hospitalization [12].

In Vietnam, eight vaccines were approved in the framework of the national COVID-19 vaccination program, including mRNA vaccines (Spikevax, Comirnaty), non-replicating viral vectors (Sputnik V, Jcovden, and Vaxzeria), inactivated virus (Covaxin, Covilo), and a protein subunit (Abdala) [13].

However, the implementation of COVID-19 vaccination has been inconsistent, leading to uneven vaccine coverage and heterologous COVID-19 vaccine combinations. The variation in vaccine availability, logistical issues with vaccine distribution, and vaccine preference or hesitancy have contributed to these inconsistencies in Vietnam and globally [14].

Despite multiple randomized clinical trials validating vaccine efficacy, there remained a need for a greater understanding of the effectiveness of booster shots and mix-and-match vaccines (a combination of different types of COVID-19 vaccines) [8,15,16]. The situation demanded a crucial filling of the knowledge gap about how the vaccine combinations work and outcomes.

In this context, our study aimed to compare the outcomes of COVID-19-vaccinated groups to an unvaccinated group of COVID-19 patients in three hospitals in Vietnam. Our method involved data collection, analysis, and modeling to identify factors associated with COVID-19 severity among hospitalized patients who had received different COVID-19 vaccine combinations. The findings of this study could have implications for health-care policymakers and practitioners in Vietnam and other countries, providing evidence-based guidance on vaccine combinations and booster dose administration to prevent and control COVID-19.

## 2. Methods

### 2.1. Study Cohort

This was an observational study involving 3102 patients admitted to 3 hospitals in Vietnam: COVID-19 Hospital (in Hoang Mai district, Hanoi), Bac Ninh Provincial Hospital (in Bac Ninh province) and Vietnam-Sweden Uong Bi Hospital (in Quang Ninh province), from Januray 2021 to May 2022. These 3102 patients were confirmed to have SARS-CoV-2 infection by at least a real-time reverse-transcriptase (RT)-PCR-positive test.

Vaccination status was classified according to the number of vaccine doses received before symptom onset in symptomatic patients and the date of COVID-19 diagnosis in asymptomatic patients. Participants were classified as unvaccinated when receiving no COVID-19 vaccine dose before the reference date, partially vaccinated if they had received one vaccine dose (PV), fully vaccinated after two vaccine doses (FV), and boosted when after three vaccine doses at least 14 days before the reference date.

### 2.2. Clinical Symptom Classification

The severity of COVID-19 was classified into five categories—no symptoms and mild, moderate, severe, and critical—according to the guidelines for diagnosis and treatment of COVID-19 issued together with Decision 4689/QD-BYT dated 6 October 2021 by the Vietnamese Ministry of Health. The clinical determination of each severity category is as follows. Patients with one of the following symptoms would be classified into the corresponding group (Table 1).

### 2.3. Data Collection Procedure

Individual patient data were obtained from the medical records, inclusive of baseline characteristics (age, sex, comorbidities), details of COVID-19 vaccination (vaccination dates and vaccine products), dates (of symptom onset, diagnosis, admission and discharge), presence of symptoms at hospital admission, laboratory test results, and treatment outcomes.

### 2.4. Statistical Analysis

The R package was used to analyze the data. All categorical variables are presented as percentages and frequencies. Fisher’s exact test or the chi-squared test was used to compare proportions. The quality variables are shown as the interquartile range (IQR) and median. The Kruskal–Wallis test was used to compare the medians. A multivariate regression analysis was built to estimate the impacts of confounders, and odds ratios (ORs) with 95% confidence intervals (CIs) were calculated. A *p*-value less than 0.05 was considered statistically significant.

Random forest (RF)- and extreme gradient boosting (XGBOOST)-based machine learning were used to identify important factors and predict patient outcomes [17].

## 3. Results

### 3.1. Participants’ Characteristics

A total of 3102 patients (median age: 46 years (IQR 38.0 years)) were involved in this study. Of them, 1475 (47%) were males and 1627 (53%) were females. The majority (1812; 57%) were older than 50. Of the 3102 patients, 1136 (37%) had at least one coexisting medical condition. The five most prevalent comorbidities were hypertension in 700 (23.0%) followed by diabetes in 469 (15%), overweight in 212 (6.8%), and heart disease, chronic kidney disease in 139 (4.5%). Patients in the vaccinated group were older than those in the unvaccinated group (48, 31.6, and 42.7 in PV, FV and boosted groups respectively vs. 29.8 in unvaccinated group). The subjects’ characteristics are presented in Table 2.

### 3.2. Types of COVID-19 Vaccine

In the studied cohort, 1342 (43.3%) patients were unvaccinated and 1760 (56.7%) were vaccinated. Out of 1760 vaccinated cases, 143 (8.1%) were PV, 1363 (77.4%) were FV, and 254 (14.5%) had received a booster dose (the third shot). In the FV group, the most commonly administered vaccine product was AstraZeneca (49%), followed by Verocell (31%), Pfizer (8.3%), Moderna (3.3%), and others (8.1%). The distribution of participants by vaccine type is presented in Table 3.

### 3.3. Association of Vaccination Status and Duration of Hospitalization or Severity of Disease

**Figure 1:**
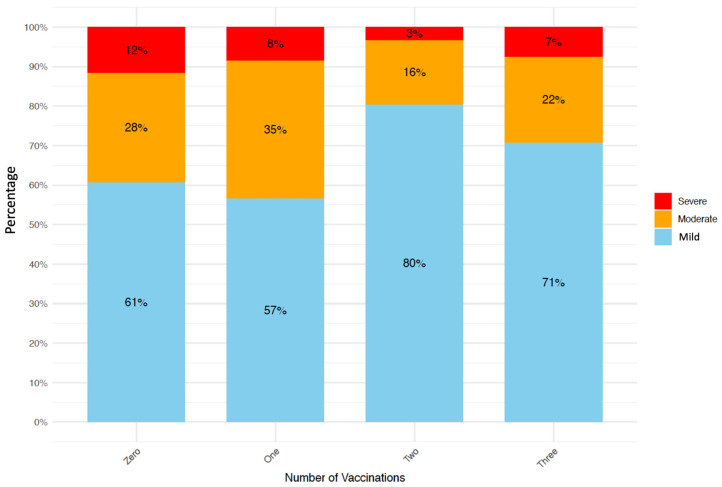
Distribution of patient conditions by vaccination status.

Figure 1 shows the distribution of patient conditions (or disease severity) by vaccination status. The unvaccinated group had a higher proportion of severe conditions than the FV group (12% vs. 3%), while mild disease was more frequent in the FV group than in the unvaccinated group (80% vs. 61%).

### 3.4. Association of Vaccination Status and Laboratory Parameters

The characteristics of some laboratory parameters between the four vaccination groups are presented in Table 4. Levels of aspartate aminotransferase (AST), ferritin, troponin T, proBNP, D-dimer, and urea were significantly higher in unvaccinated and PV patients compared to FV and boosted patients. In addition, the FV group had significantly higher lymphocyte levels and lower levels of glucose and white blood cells than other groups.

There were no differences in the other parameters among the four groups.

### 3.5. Factors Associated with Critical Illness

**Figure 2:**
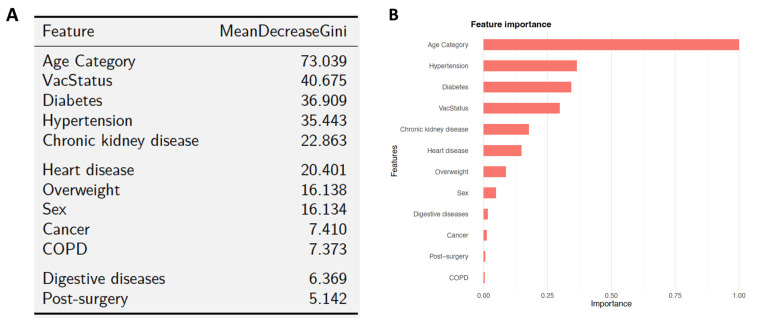
Risk factors for critical illness (identified by (**A**) random forest- and (**B**) XGBOOST-based machine learning).

The logistic regression model, including some clinical variables such as age, vaccination status, and some comorbidities, was used to identify risk factors for developing critical illness. Based on the random forest and XGBOOST analysis, age was the most crucial predictor of critical illness in patients with COVID-19, followed by vaccination status, diabetes, hypertension, chronic kidney disease, heart disease, overweight, and sex (Figure 2A,B).

**Figure 3:**
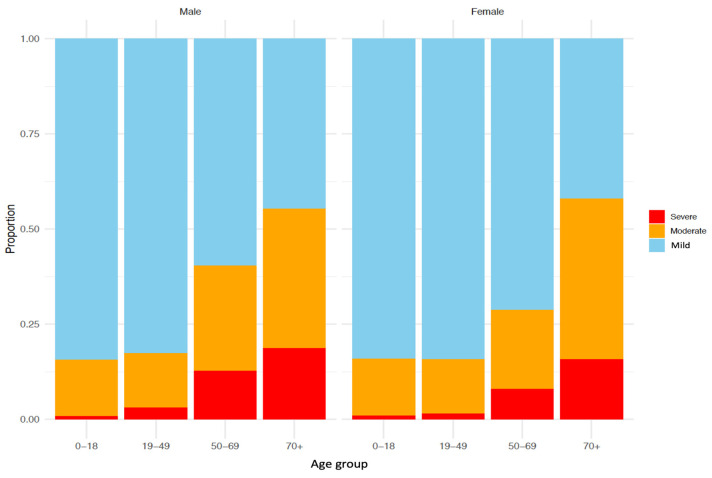
Distribution of patient conditions by age and sex.

Ordinal logistic regression and random forest-based machine learning analysis demonstrated that older age was significantly linked to both moderate and severe conditions in COVID-19 patients. Additionally, male sex and aged 50 years or older was a predictor of more severe condition (Figure 3).

### 3.6. Association of Vaccination Status and Outcomes

**Figure 4:**
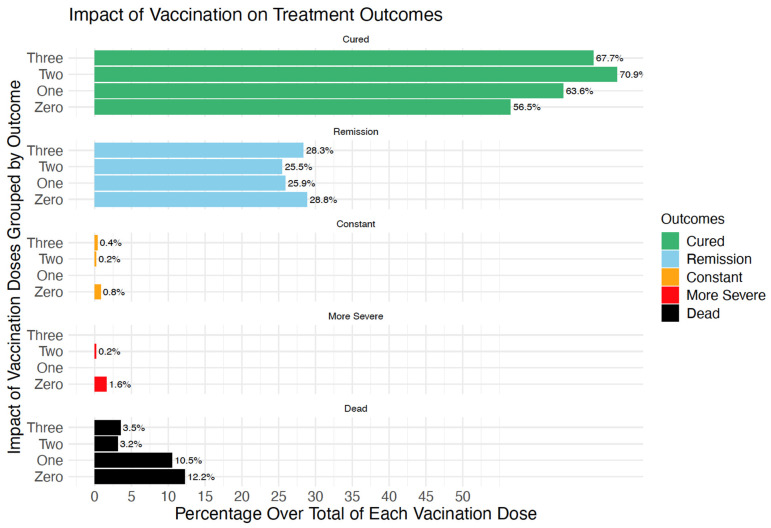
Treatment outcomes by vaccination status.

Disease outcomes are presented in Figure 4. Overall, of 3102 studied patients, 64.5% were cured, 27.1% had symptom remission, 0.4% had symptoms remaining constant, 0.8% had symptoms becoming more severe, and 7.4% died in the hospital. The vaccinated groups had higher cured rates than the unvaccinated group (64.4%, 71.3% and 67.7% for PV, FV, and boosted groups, respectively, vs. 56.8%). Worse outcomes (symptoms remaining constant, getting more severe, or death) were more frequent in the unvaccinated and PV groups than in the FV and boosted groups (12% and 10.3% vs. 3.1% and 3.5%, respectively).

**Figure 5:**
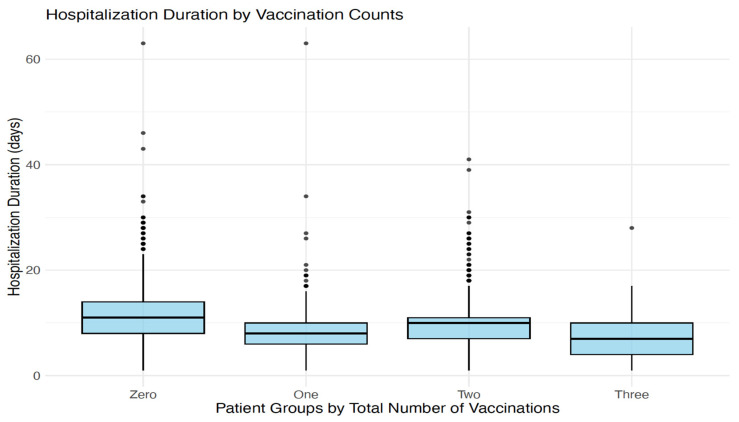
Hospitalization duration by vaccination status.

Duration of hospitalization was different by vaccination status. The mean hospitalization duration in vaccinated groups was shorter than unvaccinated groups (8, 10 and 7 days for PV, FV and boosted groups respectively vs. 11 days) (Figure 5).

## 4. Discussion

This study is one of the first in Vietnam to describe the impact of COVID-19 vaccination on treatment outcomes in COVID-19 patients. Our results showed that the rate of severe disease among Vietnamese COVID-19-vaccinated patients is lower than that of unvaccinated patients. These results are similar to those from many other studies. The vaccination has effectively improved the immune response that helps the human body cope with the disease and prevent ARD syndrome, one of the main drivers of disease severity. Vaccination also helps reduce the risk of severe disease in groups of people with underlying medical conditions [18].

Our study showed some factors prognostic of the COVID-19, such as older age, male sex, comorbidities, and vaccination status. People of all ages are susceptible to COVID-19, but the risk for moderate COVID-19 is higher in adults and severe COVID-19 is higher in adults aged 50 or older. Therefore, age 50 or older was the most important predictor of severe COVID-19. Our results were similar to the research of Statsenko et al. (2022). The risk of non-mild COVID-19 was significantly higher (*p* < 0.05) in middle-aged adults (40–64 years) and older adults (>64 years) compared to young adults [19].

A study in China reported that the mortality rate of COVID-19 patients aged older than 59 years was around 5.1 times higher than that in patients aged 30–59 years [20]. A meta-analysis showed that COVID-19 patients older than 70 years had higher risks of severe disease, intensive care, and death [21]. This study also showed that male sex and older age (>50) predicted poor condition. Males had a higher risk of being more severely affected by COVID-19. That might be related to testosterone level and differences in hormones, lifestyle, behavior, comorbidities, and socioeconomics between males and females [22].

Comorbidities are important independent factors leading to poor prognosis in COVID-19 patients [23]. These health conditions affect patient’s immunity, nutritional status, and general health, essential in overcoming infections or diseases like COVID-19. Additionally, comorbidities are commonly seen in the older population. Therefore, COVID-19 may cause more severe consequences for older persons [23]. A study by Jayaswal et al. in 2021 in India showed that diabetic patients had a 2.46-fold higher risk of severe COVID-19 [24].

Vaccination also had a positive impact on COVID-19 outcomes. For all ages, vaccination improved the immunity [25] and was associated with a reduced hospitalized fatality ratio and length of stay in hospital [18,26]. Lee et al. showed that in the USA in the whole year of 2021, the percentage of intensive care unit admissions of unvaccinated patients was higher than that of vaccinated patients (9.83% vs. 7.19%) and the length of stay in hospital of the unvaccinated group was longer than the vaccinated group (9.49 vs. 8.04 days) [27]. The indices used were hospitalized fatality ratio, length of stay in hospital, intensive care unit admission depending on severity, treatment, and comorbidities. Moreover, vaccination status helped reduce these indices. The hospitalization duration of the patients who received a booster vaccine dose was shorter than that of the other groups. The duration of hospitalization depended on many factors, such as comorbidities and severity of COVID-19 infection status. However, the effectiveness of the booster vaccine dose in reducing hospitalization should not be underestimated. Adam et al. estimated that the median number of COVID-19 vaccine booster doses needed to prevent a hospital admission was 205 and for an emergency department visit was 156 [28].

COVID-19 has been proved to be associated with changes in biomarkers such as ferritin, lactate dehydrogenase, INR, D-dimer, anemia, leukocytosis, and thrombocytopenia, and biomarkers indicative of impaired renal or hepatic function [29,30,31,32,33,34,35,36,37,38]. Ferritin was a known inflammatory biomarker and a predictor of severity level in COVID-19 [39,40,41]. The ferritin concentration in COVID-19 patients was usually higher than usual, and the concentration of the more severe patients was also higher than that of the mild patients [42]. Coagulation disorders became a prominent feature that caused various complications in COVID-19 patients.

SARS-CoV-2 damages endothelial cells by affecting angiotensin-converting enzyme 2 (ACE2) receptor 2 and transmembrane serine protease type 2 (TMPRSS2). Damage to the vascular endothelium activates endogenous and exogenous coagulation mechanisms. That is one of the pathways that causes blood-clotting disorders in people with COVID-19. Up to 95% of COVID-19 patients have coagulopathy characterized by increased D-dimer, prolonged prothrombin time, low platelet count, and other laboratory abnormalities [43]. These characteristics were also estimated for our subjects. Although there were differences among the four groups of patients, the concentration of all the biomarkers showed a decreasing trend. For example, the concentration of ferritin in many studies during the first period of the COVID-19 pandemic was significantly higher. Severe patients could reach levels of more than 2000 ng/mL, and non-severe patients could reach more than 700 ng/mL [44,45]. In our study, the concentration of ferritin in the vaccination groups was less than 500 ng/mL.

Another example was prothrombin time. In the group of severe COVID-19 patients, the prothrombin time of the deceased was longer than that of the living (15.6 (range 14.4–16.3) vs. 13.6 (range 13.0–14.3) s) [46]. With the four groups in this study, prothrombin time was shorter (the maximum variable was 12.40 s). Therefore, the vaccination improved patient condition.

Our research has some limitations. The individual patient data were collected retrospectively through the medical records, in which missing information is a limitation. The study did not fully evaluate all the factors that are likely to affect the outcome of the disease, such as underlying comorbidities, treatment measures used, or the exact period after vaccination. The available data did not allow disaggregated analysis to evaluate different vaccines’ effectiveness. The disease outcomes evaluated were only limited to several aspects without analyses of other specific clinical and paraclinical indicators that may have impact on the outcomes.

## 5. Conclusions

COVID-19 vaccination is associated with decreased mortality caused by COVID-19, severity of COVID-19, and hospitalization duration. Older age, hypertension, diabetes, heart disease, and chronic kidney disease are predictors of critical illness caused by COVID-19.

## Figures and Tables

**Table 1:** The severity categories of the COVID-19 patients.

Categories	Respiratory Symptoms					Other Symptoms
	Clinical Symptoms	Respiratory Frequency (Time/Minute)	SpO_2_ (%)	PaO_2_/FiO_2_ (mmHg)	Chest X-ray or CT Scan	
**Mild**	No difficulty breathing	<20	>96	-	Normal or minimal damage on chest X-ray	Self-service
**Moderate**	Shortness of breath exertion	20–25	94–96	-		Slow or fast pulse, normal blood pressure
**Severe**	Shortness of breath	>25	<94	200–300	Lesions more than 50%	
**Critical**	Decreased consciousness or in coma; labored or irregular breathing; respiratory acidosis	<10 or >30		<200	Lesions more than 50%	Little urine or anuria; lactate > 2 mmol/L; tachycardia, possibly bradycardia, and low blood pressure.

**Table 2:** Baseline characteristics of patients by vaccination status (number of vaccine doses).

Characteristic Variable	Overall, n = 3102	Unvaccinated Group n = 1342	1 Dose of Vaccine Group n = 143	2 Doses of Vaccine Group n = 1363	3 Doses of Vaccine Group n = 254	*p*-Value *
Age Category, N (%)						<0.001
0–18	222 (7.2)	200 (15)	2 (1.4)	20 (1.5)	0 (0)	
19–49	1476 (48)	462 (34)	44 (31)	870 (64)	100 (39)	
50–69	668 (22)	245 (18)	37 (26)	290 (21)	96 (38)	
70+	736 (24)	435 (32)	60 (42)	183 (13)	58 (23)	
Sex, N (%)						
Male	1475 (48)	618 (46)	67 (47)	661 (48)	129 (51)	0.4
Female	1627 (52)	724 (54)	76 (53)	702 (52)	125 (49)	
Comorbidities, N (%)						
0	1966 (63)	781 (58)	68 (48)	996 (73)	121 (48)	
1–3	1107 (36)	539 (40)	74 (52)	364 (27)	130 (51)	
4–11	29 (0.9)	22 (1.6)	1 (0.7)	3 (0.2)	3 (1.2)	
Diabetes, N (%)	469 (15)	236 (18)	36 (25)	147 (11)	50 (20)	<0.001
Hypertension, N (%)	700 (23)	352 (26)	49 (34)	221 (16)	78 (31)	<0.001
Chronic kidney disease, N (%)	139 (4.5)	82 (6.1)	9 (6.3)	29 (2.1)	19 (7.5)	<0.001
COPD ^1^, N (%)	42 (1.4)	20 (1.5)	3 (2.1)	11 (0.8)	8 (3.1)	0.016
Cancer, N (%)	80 (2.6)	45 (3.4)	7 (4.9)	21 (1.5)	7 (2.8)	0.004
Heart disease, N (%)	139 (4.5)	90 (6.7)	7 (4.9)	26 (1.9)	16 (6.3)	<0.001
Overweight, N (%)	212 (6.8)	86 (6.4)	10 (7.0)	96 (7.0)	20 (7.9)	0.8
Post-surgery, N (%)	31 (1.0)	21 (1.6)	3 (2.1)	4 (0.3)	3 (1.2)	0.001
Digestive diseases	37 (1.2)	20 (1.5)	3 (2.1)	11 (0.8)	3 (1.2)	0.2

* Pearson’s chi-squared test, Fisher’s exact test. ^1^ Chronic obstructive pulmonary disease.

**Table 3:** Types of vaccine product administered by vaccination group.

Variable		Overall n = 3102 ^1^	Unvaccinated Group n = 1342 ^1^	1 Dose of Vaccine Group n = 143 ^1^	2 Doses of Vaccine Group n = 1363 ^1^	3 Doses of Vaccine Group n = 254 ^1^
First shot	AstraZeneca	865 (28)	0 (0)	63 (44)	703 (52)	99 (39)
	Moderna	71 (2.3)	0 (0)	3 (2.1)	47 (3.4)	21 (8.3)
	NoVac	1342 (43)	1342 (100)	0 (0)	0 (0)	0 (0)
	Other	181 (5.8)	0 (0)	30 (21)	97 (7.1)	54 (21)
	Pfizer	158 (5.1)	0 (0)	36 (25)	87 (6.4)	35 (14)
	Sinopharm	9 (0.3)	0 (0)	1 (0.7)	5 (0.4)	3 (1.2)
	Sputnik	5 (0.2)	0 (0)	0 (0)	4 (0.3)	1 (0.4)
	Verocell	471 (15)	0 (0)	10 (7.0)	420 (31)	41 (16)
Second shot	AstraZeneca	761 (25)	0 (0)	0 (0)	669 (49)	92 (36)
	Moderna	65 (2.1)	0 (0)	0 (0)	45 (3.3)	20 (7.9)
	NoVac	1485 (48)	1342 (100)	143 (100)	0 (0)	0 (0)
	Other	164 (5.3)	0 (0)	0 (0)	110 (8.1)	54 (21)
	Pfizer	155 (5.0)	0 (0)	0 (0)	113 (8.3)	42 (17)
	Sinopharm	8 (0.3)	0 (0)	0 (0)	5 (0.4)	3 (1.2)
	Sputnik	5 (0.2)	0 (0)	0 (0)	4 (0.3)	1 (0.4)
	Verocell	459 (15)	0 (0)	0 (0)	417 (31)	42 (17)
Third shot	AstraZeneca	53 (1.7)	0 (0)	0 (0)	0 (0)	53 (21)
	Moderna	16 (0.5)	0 (0)	0 (0)	0 (0)	16 (6.3)
	NoVac	2848 (92)	1342 (100)	143 (100)	1363 (100)	0 (0)
	Other	75 (2.4)	0 (0)	0 (0)	0 (0)	75 (30)
	Pfizer	103 (3.3)	0 (0)	0 (0)	0 (0)	103 (41)
	Sinopharm	2 (<0.1)	0 (0)	0 (0)	0 (0)	2 (0.8)
	Sputnik	1 (<0.1)	0 (0)	0 (0)	0 (0)	1 (0.4)
	Verocell	4 (0.1)	0 (0)	0 (0)	0 (0)	4 (1.6)

^1^ Data presented as N (%).

**Table 4:** Comparison of laboratory parameters by vaccination group.

Patient Group by Number of Vaccinations					
Variable ^#^	Unvaccinated Group n = 1342	1 Dose of Vaccine Group n = 143	2 Doses of Vaccine Group n = 1363	3 Doses of Vaccine Group n = 254	*p* Value *
Urea (mmol/L)	5.6 (4.0–8.8)	5.7 (4.0–8.4)	4.6 (3.7–5.8)	5.2 (4.1–7.2)	<0.001
Creatinine (µmol/L)	70 (56–94)	72 (57–92)	74 (62–89)	72 (60–93)	0.076
AST (GOT) (U/L)	35 (26–54)	40 (27–68)	26 (21–35)	26 (21–43)	<0.001
ALT (GPT) (U/L)	23 (15–38)	30 (17–49)	22 (15–36)	20 (15–38)	<0.001
Glucose (mmol/L)	6.7 (5.4–9.4)	7.0 (5.8–10.5)	5.8 (5.0–7.3)	6.8 (5.3–8.9)	<0.001
Albumin (g/L)	31 (27–36)	33 (28–37)	34 (30–38)	35 (31–40)	<0.001
CRP hs (mg/dL)	3 (1–9)	3 (1–7)	3 (1–8)	2 (1–7)	0.064
Total calcium (mmol/L)	2.11 (2.04–2.21)	2.12 (2.01–2.31)	2.12 (2.09–2.13)	2.16 (1.98–2.20)	>0.9
Ion Natri (mmol/L)	135.5 (133.0–138.0)	136.0 (133.0–138.0)	137.0 (134.0–138.5)	136.0 (133.0–138.0)	<0.001
Ion Kali (mmol/L)	3.90 (3.60–4.30)	3.90 (3.60–4.20)	3.80 (3.50–4.10)	3.90 (3.60–4.20)	<0.001
Ferritin (ng/mL)	548 (263–918)	463 (198–807)	347 (143–686)	329 (187–643)	<0.001
Pro-calcitonin (ng/mL)	0 (0–2)	0 (0–0)	0 (0–1)	1 (0–6)	0.078
Troponin T (ng/L)	22 (12–54)	22 (12–37)	11 (6–18)	11 (5–31)	< 0.001
ProBNP (pg/mL)	606 (215–1378)	681 (144–1542)	148 (47–438)	206 (64–484)	<0.001
Arterial blood pH	7.42 (7.36–7.45)	7.43 (7.39–7.46)	7.42 (7.36–7.44)	7.40 (7.37–7.44)	0.2
pCO_2_ (mmHg)	35 (31–41)	35 (29–39)	36 (32–42)	36 (32–39)	0.2
pO_2_ (mmHg)	83 (65–120)	86 (64–104)	88 (67–122)	77 (64–91)	0.2
Red blood cells (T/L)	4.51 (4.06–4.96)	4.55 (4.12–4.97)	4.72 (4.35–5.13)	4.49 (4.00–5.01)	<0.001
Hemoglobin (g/L)	131 (117–142)	133 (122–146)	139 (128–150)	132 (121–146)	<0.001
Hematocrit (HCT) (L/L)	0.40 (0.36–0.44)	0.41 (0.37–0.45)	0.42 (0.39–0.46)	0.40 (0.37–0.45)	<0.001
MCV (fL)	90 (86–95)	91 (88–95)	90 (86–93)	91 (88–95)	<0.001
MCH (pg)	29.6 (28.0–31.0)	30.0 (29.0–31.0)	30.0 (28.6–31.0)	30.0 (28.0–31.0)	0.012
White blood cells (G/L)	7.0 (5.2–9.6)	6.9 (5.2–9.2)	6.3 (4.9–8.4)	7.5 (5.8–10.2)	<0.001
Lymphocytes (G/L)	1.01 (0.58–1.59)	0.91 (0.54–1.47)	1.30 (0.90–1.80)	1.10 (0.71–1.69)	<0.001
Platelet (G/L)	240 (183–311)	246 (186–326)	231 (191–289)	254 (205–317)	0.049
D-dimer (ng/mL)	650 (351–1450)	592 (293–1180)	326 (194–605)	391 (238–921)	<0.001
INR index	1.15 (1.01–1.42)	1.28 (1.16–1.49)	1.00 (0.95–1.06)	1.08 (0.98–1.38)	<0.001
Prothrombin (PT)	11.80 (11.20–12.40)	11.30 (11.20–11.80)	11.40 (11.00–12.00)	11.55 (11.13–12.00)	<0.001

^#^ Median (IQR). * Kruskal–Wallis rank-sum test.

## Data Availability

The raw data supporting the conclusions of this article will be made available by the authors on request.
